# The effectiveness of an abbreviated training program for health workers in breast cancer awareness: innovative strategies for resource constrained environments

**DOI:** 10.1186/2193-1801-2-528

**Published:** 2013-10-17

**Authors:** Miriam Mutebi, Ronald Wasike, Ahmed Mushtaq, Aideed Kahie, Stephen Ntoburi

**Affiliations:** Department of Surgery, Aga Khan University Hospital, P.O. Box 30271–00100, Nairobi, Kenya; Medical statistician, P.O. Box 19670-00100, Nairobi, Kenya

**Keywords:** Early detection, Breast cancer, Low- and middle-income, Resource allocation

## Abstract

**Background:**

Breast cancer is characterized by late presentation and significant morbidity and mortality in developing countries. Breast screening aids in early detection of breast cancer. Nurses are uniquely placed to provide advocacy and screening in a resource limited environment.

**Objectives:**

To assess the effectiveness of an abbreviated training program in breast cancer awareness on nurses at a tertiary hospital, in a resource constrained environment.

**Methods:**

Using a statistical tool, the Solomon Model, 79 nurses were identified and divided into experimental and control groups. An abbreviated training intervention in breast cancer awareness was administered to the experimental group. Pre and post test questionnaires and objective structured clinical examinations were used to determine nurses’ knowledge and practice skills before and after the abbreviated training intervention.

**Results:**

Initial scores of knowledge and practice skills related to clinical breast examination were low: Mean knowledge scores of 18 out of 25 [72%] and mean practice scores of 12.5 out of 30 [41.6%]. Significant improvement was observed following the abbreviated training intervention in both knowledge and practice skills. Knowledge scores of 22 out of 25 [88%, p = < 0.001] and practice scores of 26 out of 30 [86.6%, p=0.003]. Trained nurses were able to improve their knowledge of breast cancer from fair to good knowledge.

**Conclusion:**

There is need to increase breast awareness, both in terms of knowledge and practice skills, in nurses as a means of improving awareness among the general population and early detection of breast cancer. An abbreviated training in breast cancer awareness can improve these skills in nurses.

**Electronic supplementary material:**

The online version of this article (doi:10.1186/2193-1801-2-528) contains supplementary material, which is available to authorized users.

## Introduction

In Africa, breast cancer is characterized by a relatively young age of the affected women and an advanced stage of the disease at presentation. Newman et al. demonstrated that the overall mean age of presentation in African women is between 35 to 45 years, 10 to 15 years earlier than their Caucasian counterparts (Fregene & Newman 2005). In Kenya, the true incidence of breast cancer is underreported in the absence of a national cancer registry. It is estimated to be the commonest cause of cancer among women in Kenya comprising 23% of all malignancies in females. Data compiled by Newman and colleagues show age-standardized incidence rates (per 100,000 women) of 20.2 in Eastern Africa, of which Kenya is a part (Fregene & Newman 2005). The lack of resources and trained health personnel result in African women not having access to breast cancer screening and early diagnosis.

Screening programs have a significant impact on prognosis in breast cancer (Duffy et al. 1999, 2006). However, screening may require modification and adaptation to different resource settings. The vast majority of cancers in low income countries present in clinically advanced stages, substantially adding to the burden of the already limited cancer treatment services (Bengoa et al. 2006). Measures such as raising awareness of breast cancer and initiating cancer control programs are urgently required.

The health worker may play a critical role in promoting breast cancer awareness, more so in resource limited environments where knowledge about screening is limited among the general population. The health worker provides the critical link between the population and access to care. There is a need to standardize and assure the quality of the patient education provided and the screening evaluation performed by health workers. To achieve this end, it is necessary first to determine the level of knowledge, attitudes and practice skills of health workers in a given context.

This study assesses the utility of an abbreviated training intervention designed to increase knowledge of risk factors for breast cancer and improve practice skills of nurses in performing clinical breast examinations. The study was undertaken at the Aga Khan University Hospital in Nairobi. This tool was applied to standardize knowledge and behavior of nurses at a tertiary referral hospital.

## Results

Eighty six nurses were enrolled in the study. Four nurses declined to give consent, three nurses did not complete the day’s activities as one was called to duty and two had emergencies that prevented their participation. Seventy nine nurses subsequently had their performance analyzed. A third of the nurses enrolled (33.3%) were between 25–30 years of age (Table 1). There were approximately three times as many females. About two thirds of the respondents were married. Most nurses worked in the medical ward, followed by the surgical wards.

**Table 1:** **Characteristics of nurses enrolled in the study**

Characteristic	n	%
Age category (n=75)		
20-25	24	32
25-30	25	33.3
30-35	12	16
>35	14	18.7
Gender (n=69)		
Female	57	82.6
Male	12	17.4
Qualification (n=70)		
KRCN	53	75.7
BSc Nursing	12	17.1
Other qualification	5	7.1
Marital (n=78)		
Married	47	60.3
Single	31	39.7
Area of service (n=79)		
Medical	22	27.9
Surgical	21	26.6
Critical care	11	13.9
Paediatrics	8	10.1
Casualty	7	8.9
Out patient	6	7.6
Maternity	2	2.5
Operating Theatre	2	2.5

### Respondent practice

Seventy eight percent of respondents (61 of 78 nurses), reported having discussed breast cancer screening with their patients. The majority of the nurses had this discussion with less than 3 clients. Less than a third of nurses reported having performed a breast exam for a client. Eight in ten of the female respondents reported performing self breast examination, with more than half, doing so four or more times in the past six months.

**Figure 1:**
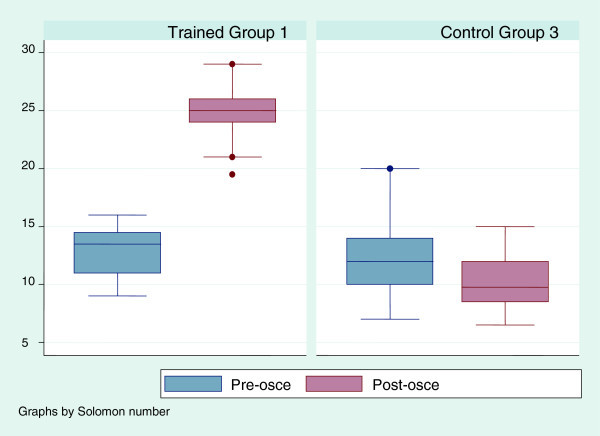
**Pre and post training osce scores in the 2 arms interventional (Group 1) and control (Group 3).** Groups 1 and 3 – Scores for OSCE before and after training.

**Figure 2:**
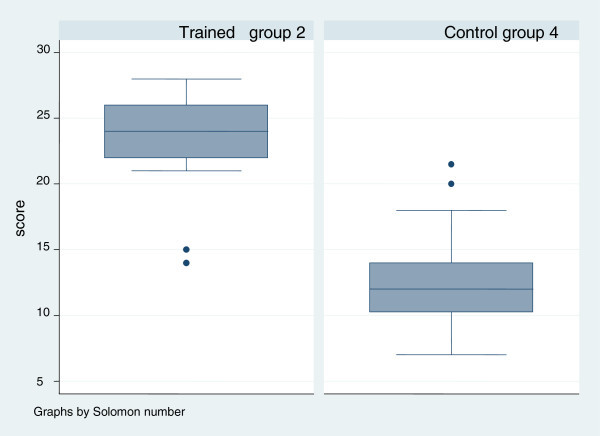
**Post training osce scores in the 2 arms interventional (Group 2) and control (Group 4).** Groups 2 and 4 – post OSCE scores.

**Figure 3:**
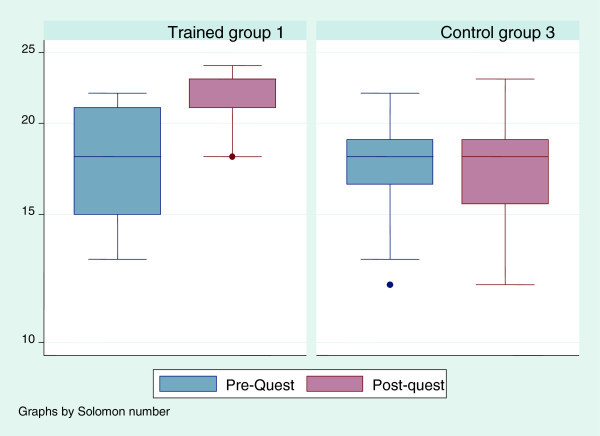
**Pre and post training questionnaire scores in the 2 arms interventional (Group 1) and control (Group 3).** Group 1 and 3 – pre and post training questionnaire results.

**Figure 4:**
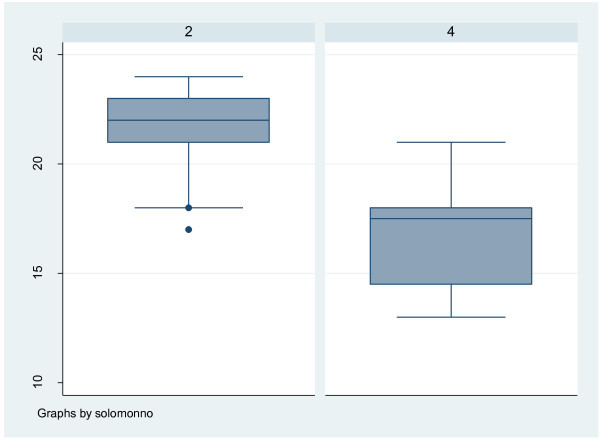
**Post training questionnaire scores in the 2 arms interventional (Group 2) and control (Group 4).** Group 2 and 4 – post questionnaire results.

Nearly all respondents (89%, 69 of 77 nurses) had previously cared for a patient with breast cancer. Nineteen of seventy eight nurses (24.6%) reported having a family member with breast cancer. Figures 1, 2, 3 and 4 show the scores for the self reported pre-questionnaire and post- questionnaire and the objective structured clinical examination by the experimental and control groups. Overall at base, the groups had comparable scores on both tests. However those in both intervention groups improved their scores to a greater extent compared to the control group (Figures 1, 2, 3 and 4).

An analysis of variance was performed to determine whether there were any differences between the four Solomon groups. There was little difference in the scores between the experimental group and the control group (p=0.64) in the pre-test questionnaire. The mean score was 19 (sd =3) and 20 (sd =3) for the experimental and control groups respectively. This shows that the two groups were comparable in both the experimental and control groups prior to intervention. The experimental groups’ mean scores were significantly higher that the control groups in the post intervention questionnaire. There was minimal difference in the mean pre-questionnaire scores between high achievers and low achievers in the different randomization groups (Additional file 1). This showed groups that were comparable at baseline. However, there was strong evidence that both high and low achievers in the experimental groups scored higher than their corresponding achievers in the control groups at the post test. A similar pattern was demonstrated in the OSCES.

Univariate regression models were fitted and the joint significance of each variable evaluated. Significant variables were then included in a multi-variate regression mode (Additional file 1). Gender and age were included in the final model a priori. The results are presented in Table 2. The groups were along Solomon groups. After controlling for other covariates, there were significant differences in the scores for the various randomization groups. All groups had higher scores than the control group that did not receive a baseline questionnaire.

**Table 2:** **Multivariate analysis of questionnaire and OSCE‡**

Variable	Questionnaire	OSCE
**Randomization group**		
GROUP 4 (CONTROL POST TEST)	Ref	Ref
GROUP 3 (CONTROL, PRE & POST TEST)	2.140**	-0.206
	(0.414 - 3.865)	(-3.094 - 2.682)
**p-value**	0.0151	0.889
GROUP 2 (TRAINED POST TEST)	4.853***	11.12***
	(3.048 - 6.658)	(7.850 - 14.39)
**p-value**	<0.00001	<0.00001
GROUP 2 (TRAINED, PRE & POST TEST)	4.051***	7.932***
	(2.281 - 5.820)	(5.004 - 10.86)
**p-value**	<0.00001	<0.00001
**IMPACT OF INTERVENTION**		
Pre-intervention	Ref	Ref
Post-intervention	1.920***	3.308***
	(1.123 - 2.716)	(0.795 - 5.821)
**p-value**	<0.00001	0.00989
**a) By age category**		
20-25	Ref	Ref
25-30	0.620	0.216
	(-0.705 - 1.944)	(-1.133 - 1.565)
	0.359	0.754
30-35	0.495	-0.670
	(-1.231 - 2.222)	(-2.531 - 1.191)
	0.574	0.480
>35	-0.267	0.139
	(-1.884 - 1.350)	(-1.512 - 1.790)
	0.746	0.869
**b) By Gender**		
Female	Ref	Ref
Male	0.0344	-1.284*
	(-1.369 - 1.438)	(-2.642 - 0.0732)
**p-value**	0.962	0.0637
**c) Cared for a patient with breast cancer**		
No	Ref	Ref
Yes	1.484	-0.204
	(-0.437 - 3.406)	(-1.807 - 1.398)
**p-value**	0.130	0.803
**d) Family history of breast cancer**		
No	Ref	Ref
Yes	0.661	-1.752**
	(-0.725 - 2.048)	(-3.257 - -0.247)
**p-value**	0.350	0.0225
**Overall mean**	13.00	10.22

in parenthesis *** p<0.01, ** p<0.05, * p<0.1 ‡ From Generalized estimating equation. Ref- reference group, category with which all others presented in the table are compared.

Counter intuitively, the intervention group that did not receive a baseline questionnaire outperformed the intervention group that received a pre-test for both questionnaires. This was unusual given the postulated priming effect of a pre-test on eventual performance. Group 1 had 4.051 (95% CI, 2.281 - 5.820; *p*<0.0001) point scores, higher than for group 3. There was a significant increase in the score from baseline to post intervention period. This table shows that age and gender were not significant factors in eventual performance. Caring for a breast cancer patient did not significantly affect performance. A family history of breast cancer did enhance the performance of the OSCE (Table 2).

A further analysis of performance based on key elements of the questionnaire and OSCE was performed. Key aspects of the questionnaire and the objective structured clinical examination were investigated. This was done to determine whether core knowledge of risk factors and critical aspects of the clinical examination had been attained during the training. It was also analyzed so as to compare the level of knowledge of these factors among the different Solomon groups. Three key factors in the questionnaire were looked at**: i) knowledge of family history as a risk factor for breast cancer ii) knowledge of age at first delivery as a risk factor iii) recognition of clinical breast examination as a screening tool.** For the clinical examination, three key areas were looked at. These were **i) recognition of breast skin and nipple changes ii) the ability to palpate all the breast quadrants iii) the ability to palpate all the axillary lymph nodes.**

In order to explore the association between performance of a task and respondent characteristics logistic regression was performed. Univariate analysis was first performed and a multivariate analysis was subsequently performed. The results presented are the odds ratio (95% confidence intervals) of being in a higher group given one’s score and controlling for other factors (Additional file 1).

The results demonstrate that both intervention groups (1 and 2) performed significantly better than the control group (3). The control group with a baseline questionnaire and OSCE (3) did not perform better on clinical task than the control group without (4). Those who were older than 25 years or who reported discussing screening with patients were also likely to examine the lymph nodes. Marital status, area of work or qualification had no significant impact on the performance of tasks. In summary, the initial scores of knowledge and practice skills related to clinical breast examination were low: Mean knowledge scores of 18 out of 25 (72%) and mean practice scores of 12.5 out of 30 (41.6%). Significant improvement was observed following the abbreviated training intervention in both knowledge and practice skills. Knowledge scores of 22 out of 25 (88%, p = < 0.001) and practice scores of 26 out of 30 (86.6%, p=0.003). Trained nurses were able to improve their knowledge of breast cancer from fair to good knowledge.

## Discussion

New strategies are required to counteract the increasing burden of breast cancer that Africa will continue to face. Hayanga et al. in a review of breast cancer in different populations demonstrated an incidence to mortality ratio of 1:5 among Caucasian American women as compared with 1.3 among African American women and 1:2 in women on the African continent (Hayanga & Newman 2007). This difference in ratios could have been the result of a lack of access to screening programs among African women. The key to changing these ratios rests in early detection and screening, to facilitate treatment of early disease. Screening methods employed in our region must address concerns about an earlier age group and a paucity of screening facilities.

In this setting, one must employ the use of traditional screening methods alongside other practical adaptations to enhance screening. With a view to achieving this end it is necessary to train health workers to increase breast health awareness in the community and to detect early breast cancer by means of breast physical examination This is in keeping with the breast global health initiative policy recommendations for limited resource settings (Anderson et al. 2008; Yip et al. 2008). Nurses’ role in raising breast cancer awareness can be expanded. Nurses have been employed to enhance adherence to screening programs and to perform CBE. In the Canadian nationa l breast cancer trials, nurse practitioners performed CBE in women who were screened (Miller et al. 2000).

Nurses are also capable of training for breast cancer screening in Kenya. WHO 2010 statistics show that whereas the doctor to population ratio in Kenya is 10 per 100,000, with many doctors practicing principally in urban areas, the nurse to population ratio, though far from ideal, is significantly higher at 120 per 100,000 (WHO 2010). Furthermore, nurses are available at most primary health centers in rural and urban areas, making them uniquely placed to reach the population. Key to the use of nurses for breast cancer screening is their training. Nurses must have a good working knowledge of the risk factors for breast cancer and be well equipped to perform CBE and offer counseling.

Several studies have demonstrated that the average nurses’ knowledge and practice skills related to breast screening is inadequate. Ahmed et al. performed a cross sectional survey of 609 female nurses in Karachi, Pakistan and found that only 35% of the nurses had 'good’ knowledge of breast cancer risk factors Ahmed et al. (2006) Ibrahim and colleagues looked at knowledge of risk factors among 550 nurses at a university hospital in Nigeria and found that 43% had 'poor’ knowledge of breast cancer risk factors (Ibrahim & Odusanya 2009). Similarly, of 431 nurses surveyed in Singapore, (Chong et al. 2002) reported that 42% of nurses had 'poor’ knowledge of breast cancer risk factors. Alkhasawneh in a review of 395 nurses in Jordan showed that knowledge of risk factors for breast cancer was 'poor’ (Alkhasawneh & Review 2007).

Nurses in the present study started off with a mean baseline knowledge of breast cancer risk factors categorized as ’fair’, irrespective of whether they were in the control or experimental arms of the study [17-18 points of 25, 68%-72%]. Knowledge improved to the 'good’ category [>22 points of 25, 88%] after training was imparted. Good knowledge [>22 points of 25, 88%] was only found in 14% of the control arm and 26% of the experimental arm before the training intervention. These findings are in keeping with those of Ahmed et al. As in this study, they utilized elements from the Stager model.

There was further analysis of the responses of the nurses based on the key elements delineated in the questionnaire. Three key elements in knowledge were evaluated. These were: age at first pregnancy, family history of breast cancer and the utility of a clinical breast examination. The results showed that 98% of nurses were able to recognize family history as a risk factor, regardless of their Solomon model assignation. There was 100% correct response in groups 1, 2 and 3 with 93% of nurses responding correctly in group 4. Similar findings were demonstrated for age at first pregnancy [96.1%].

The recognition of clinical breast examination as important for diagnosis of breast cancer was low in the pre-tested groups with only 35% to 42% of nurses correctly identifying this as a factor in screening, in both control and trained groups. This may also be due to the fact that very few nurses had actually undergone a clinical breast exam. This recognition improved to 85%-100% in groups 2 and 1 respectively. This knowledge remained low in the control groups in the post test.

In an attempt to identify criteria for selection of nurses for training in breast cancer screening, several aspects of the nurses’ bio-data were matched with their performance in the OSCE and the written test. Nurses’ previous experience of caring for breast cancer patients and their current work stations did not appear to have a positive impact on their test scores. This is in contrast to the findings by Chong et al. (2002) who undertook a cross sectional survey of 442 nurses in Singapore and observed that nurses working in a family medicine practice were more knowledgeable than those working in other areas. Admittedly, the nurses in the present study were predominantly working in internal medicine and surgery and not family medicine.

Chong in his review listed several reasons that hindered clinical breast examination including the embarrassment induced in patients as a result of examination by a male physician (Chong et al. 2002). This may have cultural implications. In Arab countries, CBE is carried out predominantly by female health workers due to cultural connotations. In the present study both male and female nurses were trained. In reviewing their overall performance it appeared females did marginally better than males. There may be greater cultural acceptability in Kenya for males to do CBE. In contrast to findings in Manchester and Jordan (Alkhasawneh & Review 2007), factors such as marital status, previous care of breast cancer patients and experience of breast self examination did not influence the knowledge level or breast examination performance of nurses in the present study. However the numbers in the present study may be too small to draw firm conclusions.

Although there was improvement in the level of knowledge as a result of training in the present study the greatest impact was on physical examination skills as shown by the result of pre and post training intervention OSCEs. This effect was sustained even at two months on follow up. The nurses commenced with mean practice scores of less than 50% and improved to mean practice scores of between 80% and 83% after training which was sustained at two months on follow-up testing. A lack of convergence of knowledge and practice skills seems to be the issue which can be overcome by regular in-service training.

Turk and Ciceklioglu, in their training of 192 nurses in fundamentals of breast knowledge and breast self exam, demonstrated the benefit of in -service training in breast cancer awareness to nurses working in the Turkish Ministry of Health (Turk et al. 2007). This was accomplished through application of an international training course on breast self examination. The lack of a control group to evaluate the effectiveness of the training was cited as a weakness of the study. We attempted to overcome this weakness in the present study by the use of the Solomon model which both eliminates the bias of pre-testing and provides a comparison group.

## Conclusion

This study shows that in service training enhances nurses’ knowledge and skills for breast cancer screening and assures accuracy of services and education provided. Pragmatic measures are required to increase breast cancer awareness and screening in resource constrained environments. Nurses are uniquely placed for these roles. This may involve utilizing trained nurses at monthly breast screening campaigns at hospitals and for community outreach. Disseminating a regional training program to unify knowledge may also contribute. This may also be extended to nurses and other health workers in training to provide knowledge about breast cancer and the importance of screening and imparting CBE skills.

## Materials and methods

This was an interventional study intending to assess the impact of training by applying an educational tool to improve knowledge and practice skills of nurses. The study aimed to assess the effects of an abbreviated standardized training of breast cancer (the 'Breast Rules’) on knowledge, attitudes and practice of nurses at a tertiary hospital in Nairobi, Kenya. The study also aimed at developing a training program/intervention for nurses and to develop a tool to test knowledge and clinical skills.

The Primary objective of the study was to determine the effectiveness of an abbreviated training program on breast screening and education for nurses at a tertiary referral hospital. Secondary objectives were to develop an assessment tool to determine baseline knowledge and skills and post training knowledge and skills and to develop a short training program to upgrade knowledge and skills that might serve a broader use in similar clinical contexts.

### Study site and participants

The study was conducted at the Aga Khan University Hospital in Nairobi. This is a tertiary referral university teaching hospital located in the East African region. The Aga Khan University Hospital, Nairobi (AKUHN) is a 254-bed private, not-for-profit, institution that provides tertiary and secondary level health care services. AKUHN is also involved in research and Postgraduate Medical Education in all major clinical specialties.

The study primarily targeted nurses. Nurses were chosen as the focus of this study because of the potentially crucial role they might play in raising breast awareness, creating a culture for free discussion of breast disease, and encouraging women to participate in regular clinical breast examination (CBE). The nurses were selected depending on their availability and shift. Inclusion criteria were nurses working at the Aga Khan University Hospital who consented to participating in the study. Exclusion criteria were nurses working in the breast clinic, the cancer/oncology units and nurses who had previously participated in a pilot survey and completed a pre-testing questionnaire on breast cancer.

Ethical approval was granted by the Research ethics committee of the Aga Khan University.

### Randomization and masking

The study was a controlled randomized educational trial. The study design employed was a randomized Solomon four- group design with two experimental groups 1 and 2, and two control groups 3 and 4 (Additional file 1). The experimental arm underwent a training intervention ('Breast rules’) and was compared to the control arm who did not receive the training intervention.

The outcomes of the study were measured using a baseline and a post intervention test questionnaire (referred to pretest and posttest respectively). It is now recognized that tests may affect the respondent’s results if the test is retaken, independent of any other interventions. Thus, with the Solomon four group design all four groups complete a posttest, but only groups 1 and 3 take a pretest (see Table 3). This design allows the researcher to assess separately the effects of the intervention and the testing (Polit & Beck 2004).

**Table 3:** **Group assignment by Solomon model**

Group	Random assignment	Observation pre-test	Experimental intervention training	Observation post-test
Experimental group 1	✓	✓	✓	✓
Experimental group 2	✓		✓	✓
Control group 3	✓	✓		✓
Control group 4	✓			✓

Nurses were invited to a one day workshop. Each eligible nurse signed an informed consent form and was allocated a study number. They were then assigned to any one of the four groups by picking - using the “blind draw” procedure - a folded paper with the group allocation written on it (Table 3). The investigators and participants were not masked to the group allocation. Half the group then had a questionnaire administered by the local trained interviewer. The entire process was coordinated by the nursing managers and nursing clinical instructors at the respective stations.

Being an experimental study, an attempt was made to avoid cross contamination of the groups. The nurses in the experimental arm [groups 1 and 2] had their training in a separate unit of the hospital far removed from the control arm nurses [groups 3 and 4]. Separate eating areas and different timings were assigned to avoid mixing of the groups during meal times and breaks. All nurses had to sign a confidentiality clause, whereby they were not to discuss the proceedings of the day amongst themselves or with any other nurses outside the study groups for the 48 hours of the intervention.

### Intervention

The experimental group received an educational intervention in the form of an abbreviated course which will henceforth be referred to as The Breast Rules course. The course was designed based on Canadian and United Kingdom’s National Health Service guidelines (Sheffield 2003), and adapted to the local setting as no local guidelines existed. The content of the training course was developed through input from experts of different disciplines including surgeons, nursing educators, radiologist, pathologist, and psychologists.

The 'Breast Rules’ course content involved an introduction to basic risk factors for breast cancer and anticipated clinical findings. The course entailed several interactive sessions and a few didactic sessions on core knowledge. The practical sessions involved small group demonstrations on effective clinical breast examination. As participants in group sessions, nurses were encouraged to discuss their specific difficulties. These sessions were facilitated by two physicians (one a surgeon), two nursing instructors and where possible a radiologist, and a social counselor depending on availability. An educational expert was consulted for the content and conduct of the training program. Different nurses with varying levels of knowledge about breast cancer and about breast screening attended this training. The training was designed to incorporate these individual needs.

### Outcomes

Two outcomes were evaluated in this study: nurses’ knowledge on breast cancer screening and clinical breast examination skills. These outcomes were evaluated using a Knowledge, Attitude and Practice (KAP) questionnaire and an Objective Structured Clinical Exam (OSCE) respectively. Three key factors in the KAP questionnaire were: (i) knowledge of family history as a risk factor for breast cancer; ( ii) knowledge of age at first delivery as a risk factor; and (iii) recognition of clinical breast examination as a screening tool. For the clinical examination (OSCE), the three key areas were: (i) recognition of breast skin and nipple changes; (ii) the ability to palpate all the breast quadrants; (iii) the ability to palpate all the axillary lymph nodes.

### Data collection

Data were collected using a knowledge questionnaire (KAP) and an objective structured clinical examination (OSCE). The administered questionnaire evaluated the knowledge, attitudes and practices of nurses towards breast cancer and breast screening. A questionnaire was developed for the study using guidelines suggested by the UK National Breast Cancer guidelines and Canadian guidelines [no local data exist]. The knowledge tool included questions incorporated from the Stager’s Comprehensive Breast Cancer Knowledge, and from a questionnaire adapted from Ahmed and colleagues (Stager 1993; Ahmed et al. 2006). Fifteen questions were developed with different scores for core knowledge. The questionnaire was pre-tested before application by a separate cohort of nurses not participating in the study and clarifications and modifications applied (Additional file 1).

Fifteen questions were incorporated into the questionnaire. Six key elements were identified and awarded a weighting of 2. The remaining nine questions carried a weighting of 1 giving a maximum score of 25. Nurses with scores of below **17**, were classified as having **poor** knowledge, **18** to **21** (69- 84%) as having **fair** knowledge and **good** knowledge indicated by a score between **22** to **25** (85-100%) points. Personal biodata, including work and personal history related especially to the breast cancer experience, were recorded.

The questionnaire determined knowledge of risk factors for breast cancer, and beliefs and attitudes towards screening. The factors analyzed were positive attitudes towards population based screening programs, familiarity with breast screening tools and attitudes towards the health worker’s role in screening. Health workers’ perception of the threat of breast cancer to women was also determined. In consideration of the younger women (age < 30 years) who routinely attended local screening sessions, basic knowledge of common breast pathology was also assessed. The knowledge assessment tool included five questions from the Stager's Comprehensive Breast Cancer Knowledge Test (8). The additional questions were formulated using international data and contextualized for the local setting. Content validity was reviewed by a breast surgeon.

Practice skills were assessed through the use of an observed structured clinical examination (OSCE) of patients. Aspects of the examination included courteous behavior, client interaction and actual breast examination skills. The nurses were invited to examine patients with breast pathology and with normal findings. 8 patients with ultrasound proven breast lumps (fibroadenomas) between 1–2 cm were used. 4 patients with normal breasts were also used. Each nurse examined 4 patients with breast lumps and two normal patients. Their method of examination was observed by two observers who assessed examination technique and the overall interaction of the nurse with the patient. The observers were trained doctors and nursing instructors. The scores of the two observers were aggregated. The observers were blinded to the nursing group assignments and to each other’s score. A final tally of scores was derived from the number of patients examined by each nurse.

Altogether fifteen elements were assessed (Additional file 1) with scores of 0 to 2 being awarded. A score of 0 was allocated for an item not performed; a score of 1 for an incompletely/inadequately performed item and a score of 2 for a completely and adequately performed item. Nurses could achieve a maximum score of 30. An 0SCE was repeated one month after the training program in order to assess retention of knowledge and skills. A sub-set of nurses who received the abbreviated training intervention were followed up in clinical practice and assessed by experienced clinical practitioners looking for any improvement in the rate of detection of breast lesions.

### Sample size

It was assumed that nurses had a 35% baseline knowledge of breast cancer risk factors and breast screening. This figure was based on data by Ibrahim and colleagues (Ibrahim & Odusanya 2009) in a university hospital in Western Africa as no local data exist. They found that the mean knowledge of risks of breast cancer and screening was 35% in 400 nurses who were assessed. A 90% increase in the knowledge, from 35% to 67% post training, was anticipated, using the 'Breast Rules’ abbreviated training module. The power of the study was set at 80% with a p-value of 0.05, to demonstrate statistical significance. A sample size included 38 providers in each arm (Total of 76 nurses).

### Statistical analysis

Several descriptive and regression analysis were conducted on the data. Data were collected and consolidated by the principal investigator and trained nursing educators. A statistician was involved as a co-investigator and assisted with data handling and analysis. Univariate analysis was undertaken to investigate participants’ knowledge and practice skills scores. Statistical comparison for qualitative and quantitative variables was carried out using analysis of variance for quantitative variables. Multivariate analysis was used to control for interaction effects.

The use of the Solomon model, attempted to analyze the effects of pretesting and the actual intervention. The use of a four group Solomon model as opposed to a standard pre and post test design, enabled analysis of variance to be performed on the different groups and the effects of pre-testing to be determined. Furthermore, the design enabled comparison with a control group. The use of the four group Solomon model enabled logistic regression analysis and multivariate analysis of group characteristics.

#### Descriptive analysis

The characteristics and work experiences of the nurses were tabulated.

#### Analysis of the KAP questionnaire and observed structured clinical exam

The mean scores for the pre and post tests were calculated. To investigate the differences in scores between the different groups adjusted for covariates we fitted generalized estimating equation (GEE), taking into account the repeated observations on the respondents. First univariable regression models were fitted and the joint significance of each variable evaluated by Wald tests. Significant variables (p<0.05) from the univariable analysis were then included in a multivariable regression. Sex and age were included in the final model a priori.

### Analysis of specific clinical examination tasks

Three clinical tasks were evaluated: 1) examination for retraction of the breast, 2) palpation of all quadrants, and 3) examination of the lymph nodes. To explore the association between performance of a task and nurses’ characteristics we performed ordinal logistic regression. Each task had three possible outcomes: 0 not done; 1 inadequately done; 2 adequately done. Univariable analysis was first conducted. Variables found significant were entered into eligible for multivariable analysis by a backward stepwise method. The analyses were not adjusted for baseline performance. The results presented are the odds ratios (with 95% confidence intervals) of achieving a higher score given the explanatory variables.

### Ethical standards

Ethical approval was sought from the University Research Ethics Committee prior to onset of the study. This study was done in compliance with the current laws of the land in Kenya.

## Authors’ information

MM is a general surgeon currently pursuing a fellowship in breast surgical oncology. MM is involved in breast cancer advocacy programs locally. RW is a breast surgeon involved in advocacy. MM and RW run a breast clinic at the oncology centre of the Aga Khan University Hospital which serves as a referral unit for the East African region. AM is professor of surgery involved in post graduate medical education. AK is a general surgeon. SN is a medical statistician.

## Electronic supplementary material

Additional file 1: **Solomon four group study model.** (DOC 249 KB)

## Abbreviations

CBE: Clinical breast examination
CME: Continuous medical education
CPT: Control group, post test
CPPT: Control Group, pre and post test training
FNAC: Fine needle aspiration cytology
KRCN: Kenya registered clinical nurse
TPT: Trained post test
TPPT: Trained, pre and post test
VP3: Vertical strip method.
