# Supplementary material for: The effectiveness of an abbreviated training program for health workers in breast cancer awareness: innovative strategies for resource constrained environments
Source: Springerplus. 2013 Oct 17;2:528. doi: 10.1186/2193-1801-2-528 (PMC3855361; doi:10.1186/2193-1801-2-528)
Supplement: Supplementary file 1 — Additional file 1: Solomon four group study model. (DOC 249 KB) [file 40064_2013_676_MOESM1_ESM.doc]

**APPENDIX I: SOLOMON FOUR GROUP STUDY MODEL**

This design is suitable when pre-testing could affect the independent variable, and thus affect measurement of its impact. A pre-test of an intervention in certain cases may affect measurement of the workshop’s effectiveness. Pre-testing would alert participants in the experimental group (those who undergo workshop training) to issues in the training and therefore they would respond more strongly than participants in the control group to such training.

Researchers using the Solomon four-group design identify two experimental and two control groups, and pre-test one experimental group and one control group only. This enables them to isolate the effects of pre-testing and intervention. The table below illustrates how this design works.

**Steps in the Solomon Four-Group Design**

| **Group** | **Random Assignment** | **Observation**  **Pre-test** | **Experimental Treatment**  **Training** | **Observation**  **Post-test** |
| --- | --- | --- | --- | --- |
| Experimental group 1 |  |  |  |  |
| Control group 1 |  |  |  |  |
| Experimental group 2 |  |  |  |  |
| Control group 2 |  |  |  |  |

To determine if the effect of the pre-test had any effect, researchers compare groups 1 and 3 or 2 and 4. The Solomon four-group design is useful as it allows the results to be generalized because random assignment, observation, the experimental treatment, and observation are applied to all four groups. In the study, if the pre-test sensitizes students, both of the pre-test groups (1 and 2) will have higher scores than the groups that did not undergo pre-testing (3 and 4). If the pre-test motivates study participants who received the experimental intervention (groups 1 and 3), group 1 will have higher post-test scores than group 3.

A Solomon four-group study seeks to assess the effect of treatment, the effect of pre-testing, and the interaction between pre-testing and intervention.

**APPENDIX II: BREAST RULES TRAINING GUIDE**

**Training group**

Confirm inclusion criteria

Get signed informed consent

Fill in study subject number

**Control group**

Confirm inclusion criteria

Get signed informed consent

Fill in study subject number

**BREAST RULES CURRICULUM**

An abbreviated training in breast care awareness

**Training objectives:**

At the end of the training, the nurse should:

Demonstrate proficiency in clinical breast examination

Know practical algorithm for dealing with breast lumps in different age groups

Know basic risk factors for breast cancer

Know proper mode of evaluation of a breast lump and proper referral channels

**Breast rules program:**

**Program outline:**

Introduction, consent signing

Pre-test evaluation and observed OCSE

Introduction and outline of course objectives

Session I – Didactic lectures

Session II- Didactic lectures

**Tea break**

Session III- Didactic lectures

Session IV- Demonstration of breast examination

**Lunch break:**

Practical interactive session

Post - test evaluation and Observed OSCE

Vote of thanks, feedback slips.

**Curriculum structure**

**Selection of trainees**

Nurses will be selected from the medical, surgical, critical care, theatre and accident and emergency areas of the hospital. The study will take place over 48 hours with 30 nurses being trained on one day and another 30 on the next day. The nurses are blinded to the training that they shall undertake and will be signed off duty to attend an educational workshop. It will be impossible to blind the nursing managers who organize nursing duties and will indicate the nurses who will be released for this exercise

The experimental arm of the nurses will be trained on the first day and the control arm trained on the next day. Attempts to avoid cross contamination of the groups will be made by ensuring no communication between the two groups and by asking the nurses to sign a ‘no disclosure’ policy for the 48 hours of the study. The nursing care managers will ensure no interaction between the nurses on respective days in terms of coinciding duties. It proved logistically impossible to release 60 nurses out of the 300 at any time to take part in this study, hence the need for a rapid temporal control over 48 hours and relative seclusion of the two groups to minimize cross contamination of the two groups.

The nurses will undergo a series of didactic lectures and a practical training session. Half of the experiment group will be subjected to a pre-test questionnaire and practical evaluation. All 30 trained nurses will undergo a post test questionnaire and demonstration.

The nurses in the control group will undergo a similar exercise where half the group shall be subjected to a pre-test and questionnaire. The thirty nurses shall then undergo series of non related sessions to cover the same time they would have used for the breast training session. All thirty nurses will undergo a post test evaluation.

This experiment incorporates the use of a Solomon model which is a four arm tool designed to evaluate the efficacy of an interventional tool. The four arm design described above helps to ensure self validation of a given tool. This model aims to provide both internal and external validity of a given intervention. This model minimizes the inherent biases that a pretest evaluation could predispose to.

True randomization demands that all the nurses in the respective units have an equal chance of being involved in the study. In this instance true randomization was not possible as the availability of the nurse depended on their shift and available duties. It was not possible to release 60 nurses at a single time, thus the two groups had to be evaluated over the two respective days. Each nurse on a given day will have an equal chance of falling into one of the two Solomon groups being evaluated on the day in question. This is thus a quasi-experimental study and not a true experiment.

**Training and sessions**

Structure:

A pretest questionnaire will be administered to two Solomon groups which will assess their baseline knowledge, attitudes and practice.

They shall then be subjected to a practical evaluation where they shall palpate five patients with varying sizes of lumps and their approach observed and assessed.

Training will involve didactic sessions (see program above) followed by an interactive 30 minute sessions for questions and answers.

The afternoon session will involve a practical demonstration of breast examination skills by content experts. A demonstration video will be shown initially to the thirty nurses. This will then involve practical demonstration, with small group clusters of five. One surgeon and one trained nursing trainer will coordinate this session. Practical difficulties and concerns will be aired at this session.

The nurses will then be subjected to a post test questionnaire and repeat OSCE.

**APPENDIX III: QUESTIONNAIRE**

**HEALTH WORKER SURVEY**

Participant number: _____________________________

Questionnaire

Age group:

20 – 25 25-30 30-35 35-45 45-50

Health worker category; Nurse Qualification____________________

Gender: Male Female

Years of practice _____________________________________________

Marital status: Single Married Divorced

Unit of practice:

Medical Surgical Critical care OPD

Other (specify) ______________________________________________

Do you discuss the significance of breast screening to your clients?

Yes No

If yes, of last 10 clients seen before today, how many did you mention it to? 1-3 4-6

Do you routinely perform breast exam on you clients? Yes No

If yes, how many times in the last six months ______________________

Have you ever had a clinical breast examination

performed by your doctor? Yes No

Have you ever cared for a patient with breast cancer? Yes No

What are the risk factors for breast cancer?

| **Question** | **Yes** | **Not sure** | **No** |
| --- | --- | --- | --- |
| Breast cancer is more common in older women than younger women (1mark) |  |  |  |
| The incidence of breast cancer in Kenya is on the decline  (1mark) |  |  |  |
| Family history of breast cancer increases one’s risk of getting breast cancer (2 mark) |  |  |  |
| Family history of ovarian cancer increases one’s risk of getting breast cancer ( 1mark) |  |  |  |
| Breast feeding increases the chances of getting breast cancer (2 mark) |  |  |  |
| Alcohol consumption increases one’s risk of getting breast cancer (1mark) |  |  |  |
| Smoking increases one’s risk of getting breast cancer (1mark) |  |  |  |
| Patients with breast cancer will all die from their disease (1 mark) |  |  |  |
| Clinical breast examination may detect breast cancer (2marks) |  |  |  |
| Mammography can detect small breast cancerous lesions (2 marks) |  |  |  |
| Breast cancer is a disease of socially disadvantaged individuals (1mark) |  |  |  |
| Having children decreases your risk of breast cancer(1mark) |  |  |  |
| Irritation of the bra can cause breast cancer (1 mark) |  |  |  |
| In some women, being overweight may increase risk of breast cancer (1 mark) |  |  |  |
| Use of oral contraceptive pills may increase a woman’s risk of getting breast cancer (2 marks) |  |  |  |
| Most breast lumps are cancerous (1 mark) |  |  |  |
| A lady who bears her first child after 30 years is more likely to develop breast cancer (2 marks) |  |  |  |
| Breast cancer is a communicable disease (1mark) |  |  |  |
| Breast cancer can be the result of a curse/evil eye (1mark) |  |  |  |
| Total 25 |  |  |  |

**APPENDIX IV: OBJECTIVE STRUCTURED CLINICAL EXAMINATION**

| Items | Not attempted (0) | Attempted, incomplete (1) | Done well (2) |
| --- | --- | --- | --- |
| Show courtesy |  |  |  |
| Adequate exposure |  |  |  |
| Examines patient sitting up |  |  |  |
| Asks patient to raise arm |  |  |  |
| Asks patient to press on hips |  |  |  |
| Comments on symmetry/scars |  |  |  |
| Comments on retraction/discharge/dimpling |  |  |  |
| Inspects |  |  |  |
| Starts examining normal breast |  |  |  |
| Palpates all quadrants/Axillary tail |  |  |  |
| Palpates all lymph node groups |  |  |  |
| Palpates supraclavicular nodes/liver |  |  |  |
| Discussion of findings with patient |  |  |  |
| Reassuring/counselling patient |  |  |  |
| Covers patient and thanks him/her |  |  |  |
|  |  |  |  |
| Total |  |  |  |

**APPENDIX 5 : ANALYSIS**

**Table 4: A**nalysis of variance of questionnaire

|  | N | | Mean | | SD | | MSE | | F | | P | |  |
| --- | --- | --- | --- | --- | --- | --- | --- | --- | --- | --- | --- | --- | --- |
| **Pre-questionnaire** |  | |  | |  | |  | |  | |  | |  |
| Experimental 1 | 19 | | 18 (72%) | | 3 | | 2.69 | | 0.22 | | 0.64 | |  |
| Control 2 | 20 | | 18 (72%) | | 3 | |  | |  | |  | |  |
| **Post –questionnaire** |  | |  | |  | |  | |  | |  | |  |
| Experimental 1 | 18 | | 22 (88%) | | 2 | |  | |  | |  | |  |
| Experimental 2 | 20 | | 22 (88%) | | 2 | | 2.03 | | 31.11 | | <0.0001 | |  |
| Control 1 | 24 | | 18 (72%) | | 2 | |  | |  | |  | |  |
| Control 2 | 16 | | 17 ( 68%) | | 2 | |  | |  | |  | |  |
| **Pre questionnaire (high achievers)** |  | |  | |  | |  | |  | |  | |  |
| Experimental1 | 5 | | 22 (88%) | | 1 | | 0.56 | | 0.43 | | 0.54 | |  |
| Control1 | 3 | | 21 (84%) | | 1 | |  | |  | |  | |  |
| **Pre –questionnaire(low achievers)** | |  | |  | |  | |  | |  | |  | |
| Experimental | 8 | | 18 (72%) | | 1 | | 1.07 | | 0.35 | | 0.56 | |  |
| Control | 12 | | 18( 72%) | | 1 | |  | |  | |  | |  |
| **Post questionnaire(high achievers)** |  | |  | |  | |  | |  | |  | |  |
| Experimental1 | 5 | | 23 (92%) | | 1 | | 1.46 | | 4.37 | | 0.09 | |  |
| Control1 | 8 | | 17 (68%) | | 1 | |  | |  | |  | |  |
| **Post –questionnaire(low achievers)** |  | |  | |  | |  | |  | |  | |  |
| Experimental1 | 8 | | 23(92%) | | 1 | | 1.52 | | 51.7 | | <0.0001 | |  |
| Control1 | 12 | | 18(72%) | | 2 | |  | |  | |  | |  |

N=observations, SD=standard deviation; MSE =model standard error; F=f-statistic, p=p value given degrees of freedom and f statistic; high achiever defines as score>17 on pre questionnaire while low achievers define as >17

The results of ANOVA for the OSCE score are presented in table 5. There were no significant differences in the OSCE scores, before the intervention, between the control and experimental groups. The experimental group however achieved high scores in the post training intervention test. Those who had taken the test previously performed marginally better in the experimental group but worse in the control group.

Table 5: Analysis of variance for objective structured clinical examination.

|  | N | Mean | SD | MSE | F | P |
| --- | --- | --- | --- | --- | --- | --- |
| **Pre-OSCE** |  |  |  |  |  |  |
| Experimental | 17 | 13 | 2.3 | 2.86 | 0.81 | 0.38 |
| Control | 21 | 12 | 3.3 |  |  |  |
| **Post – OSCE** |  |  |  |  |  |  |
| Experimental1 | 18 | 25 | 2.6 |  |  |  |
| Experimental2 | 19 | 24 | 3.8 | 3.16 | 114.4 | <0.000 |
| Control 1 | 24 | 10 | 2.3 |  |  |  |
| Control2 | 16 | 13 | 4 |  |  |  |
| **Pre– OSCE (high achievers)** |  |  |  |  |  |  |
| Experimental | 5 | 14 | 1.2 | 2.61 | 1.63 | 0.25 |
| Control | 3 | 12 | 4.2 |  |  |  |
| **Pre – OSCE (low achievers)** |  |  |  |  |  |  |
| Experimental | 7 | 12 | 1.9 | 2.26 | 0.02 | 0.88 |
| Control | 12 | 11 | 2.4 |  |  |  |
| **Post – OSCE (high achievers)** |  |  |  |  |  |  |
| Experimental | 4 | 26 | 1.3 | 2.27 | 78.6 | 0.0003 |
| Control | 3 | 11 | 3.3 |  |  |  |
| **Post – OSCE (low achievers)** |  |  |  |  |  |  |
| Experimental | 8 | 24 | 3.1 | 2.74 | 130.9 | <0.00001 |
| Control | 12 | 10 | 2.5 |  |  |  |

N=observations, SD=standard deviation; MSE =model standard error; F=f-statistic, p=p value given degrees of freedom and f statistic; high achiever defined as pre-questionnaire score>17 while low achievers define as pre-questionnaire score<17.

Table 6: Multivariate analysis of questionnaire and OSCE‡

| **Variable** | **Questionnaire** | **OSCE** |
| --- | --- | --- |
| **Randomization group** |  |  |
| GROUP 4(CONTROL POST TEST) | Ref | Ref |
| GROUP 3(CONTROL, PRE& POST TEST) | 2.140** | -0.206 |
|  | (0.414 - 3.865) | (-3.094 - 2.682) |
| p-value | 0.0151 | 0.889 |
| GROUP 2(TRAINED POST TEST) | 4.853*** | 11.12*** |
|  | (3.048 - 6.658) | (7.850 - 14.39) |
| p-value | <0.00001 | <0.00001 |
| GROUP 2(TRAINED, PRE & POST TEST) | 4.051*** | 7.932*** |
|  | (2.281 - 5.820) | (5.004 - 10.86) |
| p-value | <0.00001 | <0.00001 |
| **IMPACT OF INTERVENTION** |  |  |
| Pre-intervention | Ref | Ref |
| Post-intervention | 1.920*** | 3.308*** |
|  | (1.123 - 2.716) | (0.795 - 5.821) |
| p-value | <0.00001 | 0.00989 |
| **a) By age category** |  |  |
| 20-25 | Ref | Ref |
| 25-30 | 0.620 | 0.216 |
|  | (-0.705 - 1.944) | (-1.133 - 1.565) |
|  | 0.359 | 0.754 |
| 30-35 | 0.495 | -0.670 |
|  | (-1.231 - 2.222) | (-2.531 - 1.191) |
|  | 0.574 | 0.480 |
| >35 | -0.267 | 0.139 |
|  | (-1.884 - 1.350) | (-1.512 - 1.790) |
|  | 0.746 | 0.869 |
| **b) By Gender** |  |  |
| Female | Ref | Ref |
| Male | 0.0344 | -1.284* |
|  | (-1.369 - 1.438) | (-2.642 - 0.0732) |
| p-value | 0.962 | 0.0637 |
| **c) Cared for a patient with breast cancer** |  |  |
| No | Ref | Ref |
| Yes | 1.484 | -0.204 |
|  | (-0.437 - 3.406) | (-1.807 - 1.398) |
| p-value | 0.130 | 0.803 |
| **d) Family history of breast cancer** |  |  |
| No | Ref | Ref |
| Yes | 0.661 | -1.752** |
|  | (-0.725 - 2.048) | (-3.257 - -0.247) |
| p-value | 0.350 | 0.0225 |
| Overall mean | 13.00 | 10.22 |

in parenthesis *** p<0.01, ** p<0.05, * p<0.1 ‡ From Generalized estimating equation. Ref- reference group, category with which all others presented in the table are compared.

Table 7: Multivariate ordered logistic regression for performance of tasks.

|  |  |  |  |
| --- | --- | --- | --- |
| VARIABLES | Identifies changes | Palpates all quadrants | Palpates all lymph node groups |
|  |  |  |  |
| **Randomization group** |  |  |  |
|  |  |  |  |
| GROUP 3 | 1.327 | 3.030 | 3.987 |
|  | (0.191 - 9.207) | (0.374 - 24.58) | (0.407 - 39.07) |
| p-value | 0.775 | 0.299 | 0.235 |
| GROUP 2 | 30.73*** | 123.8*** | 45.66*** |
| ## | (2.971 - 317.8) | (6.038 - 2,537) | (3.475 - 600.1) |
| p-value | 0.00406 | 0.00177 | 0.00364 |
| GROUP 1 | 21.58** | 27.59** | 869.3*** |
|  | (1.770 - 263.1) | (1.829 - 416.2) | (21.22 - 35,617) |
| p-value | 0.0161 | 0.0166 | 0.000354 |
| **Age category** |  |  |  |
| 20-25 | Ref | Ref | Ref |
| 25-30 | 5.294** | 0.821 | 79.69*** |
|  | (1.021 - 27.46) | (0.151 - 4.471) | (6.910 - 919.0) |
|  | 0.0472 | 0.820 | 0.000449 |
| 30-35 | 0.304 | 0.198 | 24.33** |
|  | (0.0399 - 2.315) | (0.0231 - 1.694) | (1.703 - 347.6) |
|  | 0.250 | 0.139 | 0.0186 |
| >35 | 2.189 | 0.0645** | 24.04*** |
|  | (0.393 - 12.19) | (0.00714 - 0.583) | (2.311 - 250.1) |
|  | 0.371 | 0.0147 | 0.00779 |
| **Gender** |  |  |  |
| Female | Ref | Ref | Ref |
| Male | 0.275 | 0.0859** | 0.394 |
|  | (0.0358 - 2.109) | (0.00907 - 0.814) | (0.0534 - 2.902) |
|  | 0.214 | 0.0324 | 0.360 |
| **Qualification** |  |  |  |
|  |  |  |  |
| Bsc nursing | 0.414 | 0.485 | 0.925 |
|  | (0.0773 - 2.219) | (0.0751 - 3.125) | (0.124 - 6.895) |
|  | 0.303 | 0.446 | 0.940 |
| Other nursing qualification | 0.356 | 0.439 | 0.548 |
|  | (0.0310 - 4.088) | (0.0238 - 8.064) | (0.0480 - 6.255) |
|  | 0.407 | 0.579 | 0.628 |
| Marital status |  |  |  |
| Single | Ref | Ref | Ref |
| Married | 0.792 | 0.446 | 2.825 |
|  | (0.220 - 2.859) | (0.0935 - 2.129) | (0.673 - 11.85) |
|  | 0.722 | 0.311 | 0.156 |
| Area of service |  |  |  |
| Non surgical |  |  |  |
| Surgical | 0.674 | 0.565 | 1.151 |
|  | (0.142 - 3.207) | (0.0861 - 3.701) | (0.177 - 7.482) |
|  | 0.620 | 0.551 | 0.883 |
| Discussed Screening |  |  |  |
| No |  |  |  |
| Yes | 1.458 | 1.254 | 56.33** |
|  | (0.176 - 12.04) | (0.152 - 10.31) | (2.577 - 1,231) |
|  | 0.726 | 0.833 | 0.0104 |

Table 8: Performance of various clinical tasks by Solomon groups

| **Risk factor recognition** | GROUP 1  (%) | GROUP 2  (%) | GROUP 3  (%) | GROUP 4  (%) |
| --- | --- | --- | --- | --- |
| **I) family history of breast cancer** |  |  |  |  |
| *Pre-intervention(n=40)* |  |  |  |  |
| Incorrect | 0 |  | 0 |  |
| Correct | 19(100) |  | 20(100) |  |
| *Post-intervention(n=76)* |  |  |  |  |
| Incorrect | 0 | 0 | 0 | 1(6.2) |
| Correct | 18(100) | 19(100) | 23(100) | 15(93.8) |
| **2) Age at first pregnancy** |  |  |  |  |
| *Pre-intervention(n=39)* |  |  |  |  |
| Incorrect | 0 |  | 2(10) |  |
| Correct | 19(100) |  | 18(90) |  |
| *Post-intervention(n=77)* |  |  |  |  |
| Incorrect | 0 | 1(5) | 2(8.7) | 0 |
| Correct | 18(100) | 19(95) | 21(91.3) | 16(100) |
| **3) Recognized importance of clinical breast examination** |  |  |  |  |
| Pre-intervention(n=39) |  |  |  |  |
| Incorrect | 11(57.9) |  | 13(65) |  |
| Correct | 8(42.1) |  | 7(35.0) |  |
| Post-intervention(n=77) |  |  |  |  |
| Incorrect | 0 | 3(15) | 15(65.2) | 15(93.8) |
| Correct | 18(100) | 17(85.0) | 8(34.8) | 1(6.25) |
| **1) Recognized breast skin changes** |  |  |  |  |
| *Pre-intervention(n=39)* |  |  |  |  |
| not attempted | 8(47.1) |  | 16(76.2) |  |
| attempted incomplete | 5(29.4) |  | 2(9,5) |  |
| done well | 4(23.5) |  | 3(14.3) |  |
| *Post-intervention(n=77)* |  |  |  |  |
| not attempted | 5(27.8) | 4(21.0) | 16(66.7) | 10(62.5) |
| attempted incomplete | 1(5.7) | 4(21.0) | 6(25.0) | 3(18.8) |
| done well | 12(66.7) | 11(57.9 | 2(8.3) | 3(18.8) |
| **2) Palpated all breast quadrants** |  |  |  |  |
| *Pre-intervention(n=36)* |  |  |  |  |
| not attempted | 4(23.5) |  | 1(5.3) |  |
| attempted incomplete | 8(47.1) |  | 10(52.6) |  |
| done well | 5(29.4) |  | 8(42.1) |  |
| *Post-intervention(n=77)* |  |  |  |  |
| not attempted | 2(11.1) | 0(0) | 1(4.2) | 1(6.25) |
| attempted incomplete | 2(11.1) | 2(10.5) | 16(66.7) | 8(50.0) |
| done well | 14(77.2) | 17(89.5) | 7(29.2) | 7(43.8) |
| **3) Palpated all lymph node groups** |  |  |  |  |
| *Pre-intervention(n=38)* |  |  |  |  |
| not attempted | 12(70.6) |  | 15(71.4) |  |
| attempted incomplete | 5(29.4) |  | 5(23.8) |  |
| done well | 0(0) |  | 1(4.8) |  |
| *Post-intervention(n=76)* |  |  |  |  |
| not attempted | 2(11.1) | 3(15.8) | 16(66.7) | 10(66.7) |
| attempted incomplete | 3(16.7) | 3(15.8) | 6(25.0) | 4(26.7) |
| done well | 13(72.2) | 13(68.4) | 2(8.3) | 1(6.7) |
